# Temporal and spatial variations of net anthropogenic nitrogen inputs (NANI) in the Pearl River Basin of China from 1986 to 2015

**DOI:** 10.1371/journal.pone.0228683

**Published:** 2020-02-10

**Authors:** Xia Cui, Caizhu Huang, Jiapeng Wu, Xiaohan Liu, Yiguo Hong

**Affiliations:** 1 School of Economics and Statistics, Guangzhou University, Guangzhou, China; 2 Key Laboratory for Water Quality and Conservation of the Pearl River Delta, Ministry of Education, Institute of Environmental Research at Greater Bay, Guangzhou University, Guangzhou, China; Chinese Research Academy of Environmental Sciences, CHINA

## Abstract

Human activities have greatly influenced the natural nitrogen cycle, causing dramatic degradation of ecosystem function. Net anthropogenic nitrogen input (NANI) is an important factor contributing to the impact of human activities on the regional nitrogen cycle. Here, we analyzed the temporal and spatial variation of NANI in the Pearl River Basin of China between 1986 to 2015, and found that the total amount of NANI significantly increased from 3,362.25 kg N km^-2^ yr^-1^ to 8,071.15 kg N km^-2^ yr^-1^. Application of nitrogen fertilizers was the largest component of NANI in the Basin, accounting for 55.53% in the total NANI, followed by food/feed net nitrogen input (21.26%), atmospheric nitrogen deposition (12.95%), and crop nitrogen fixation (10.26%). Over the last three decades, nitrogen inputs from atmospheric nitrogen deposition have become the second largest source of NANI due to rapid industrialization and urbanization in the region. Regression analysis showed that the rapid growth of both GDP and population density were the main contributors to the increase of NANI. In addition, the increase in the number of red tides in the Pearl River Estuary was strongly correlated with NANI discharge (*R*^*2*^ = 0.90, *p*<0.01), suggesting the NANI’s eutrophication effect. In total, this study provides a quantitative understanding of the temporal and spatial variations of NANI in the Pearl River Basin as well as the effects of NANI on estuarine waters, and offered key information for developing an integrated strategy for watershed nitrogen management.

## 1 Introduction

Nitrogen (N) is one of the most abundant elements in nature, controlling the evolutionary processes and biodiversity of ecosystems [1–3]. With the increasing intensity of human activities, N concentrations and flux in Basins increased steadly, becoming one of the main factors in the degradation of river ecosystems as well as the eutrophication of water bodies such as lake banks, estuaries, and coasts [4–8]. Previous studies showed that a substantial amount of anthropogenic N was transported to estuarine and coastal waters via river flow[8]. In order to controling watershed N pollution, it should be important to illuminate the input flux of sources of anthropogenic N and the effects on N export[9–14]. As the most populous developing country in the world, China has achieved a great economic development. At same time, the problems of population expansion and resource shortage became more and more prominent. Thus, rapid development had strongly negative impacts on the environment. In particular, more than 80% of waterbodies in China are highly eutrophic due to large amounts of N input [15–17]. The input of human N sources in the Yangtze River, Yellow River, and Pearl River have exceeded the natural N fixation[18, 19]. According to the China’s Bulletin of Environmental Quality of Coastal Waters in 2016, the pollution of surface waters was serious. Among the 1940 sections of national water quality monitoring, Grade I-II quality waters (the lower the grade, the better the water quality) only accounted for 39.9%, while 27.9% waters reached Grade-III quality standards and 32.3% of waters were belonged to Grade IV, V, and worse than Grade V [20].

In particular, the Pearl River Basin is one of most contaminative regions in China, with inorganic N and active phosphate as the most serious pollutants [20]. An excess of N caused by human activities has a negative impact on environmental health and sustainability of coastal waters [7, 21, 22]. Therefore, it is very important to understand and characterize the temporal and spatial variations of N in the Pearl River Basin and their effects on estuarine waters.

Previous studies have shown that net anthropogenic N inputs (NANI) are an effective index to quantify the combined effects of N inputs from human activities [23]. NANI is widely used to estimate the N input at both watershed and regional scales. Several regional NANIs have been estimated across the world, including the western and northeastern U.S. [24–26], Europe [27], China [28, 29] and India [30]. The typical NANI model consists of four components: N fertilizer application, food/feed net N input, atmospheric N deposition, and crop N fixation [23]. Using this model, several studies have calculated the NANI input into different Basins. To determine the effective impact of N input estimates on the predicting of riverine N export, nine separate NANI models used for each of 18 Lake Michigan catchments and demonstrated that different budgeting models can significantly influence the estimation of N input[31]. Gao et al. [3] estimated the socioeconomic indexes of sub-basins by an area-weighted method and land use-weighted method, and then obtained the NANI for the Dianchi Lake in China from 2000 to 2010 and the two different weighted methods led to about a 15% difference. The study about the temporal and spatial variations of NANI in the Dongting Lake Basin of China suggested that the government should focus on controlling the input of upstream pollutants and the loss of non-point source nutrients from the land [17]. Chen et al. [14] studied the variation of NANI in the Yangtze River Basin in China from 1980 to 2012 and its regression relationship with dissolved inorganic N (DIN), indicating that NANI in the southern part of the Basin has more than tripled over the past 30 years. They also found that NANI contributed to 37~66% of the river DIN export in the Yangtze River Basin.

In the last 30 years, with globalization of China, the Pearl River Basin has played an ever-increasing role in the development of the regional economy. According to 2015 statistics the Pearl River Basin, despite accounting for just 5 percent of the total Chinese territory and 8.22 percent of its population, generated more than 100 billion dollar accounting for 11.39 percent of the national Gross Domestic Product (GDP). Furthermore, it is the primary water supply for human consumption, agriculture, hydropower, and shipping in southern China. However, the Pearl River Estuary has been eutrophic since the 1970s, resulting in the blooms of harmful algae as well as environmental and economic losses [32–35]. However, there are few studies on the evaluation of N sources from human activities in the Pearl River Basin. A recent study estimated DIN and dissolved inorganic phosphorus (DIP) between 1970~2050 using the Global NEWS-2 model, and pointed out that it was difficult to identify the key areas of N source input by estimating NANI from provincial scale data [36]. In order to improve the management of N in the watershed, it is necessary to estimate NANI at the level of the Pearl River Basin, as well as investigate the N source emission patterns of sub-basins.

In this study, we focused on the temporal and spatial variation of NANI into the Pearl River Basin of China from 1986 to 2015. In first, we analyzed the temporal and spatial variation of NANI across the entire Basin and calculated the contribution of different N input sources. Then, we analyzed the relationships between NANI and population or GDP in order to revealing the impact of economic development, as well as identifying the main NANI sources. Together, this study would give us a deep insight into the temporal and spatial evolution of NANI and provide suggestions for controlling N pollution in the Pearl River Basin.

## 2 Materials and methods

### 2.1 Study area

**Fig 1:**
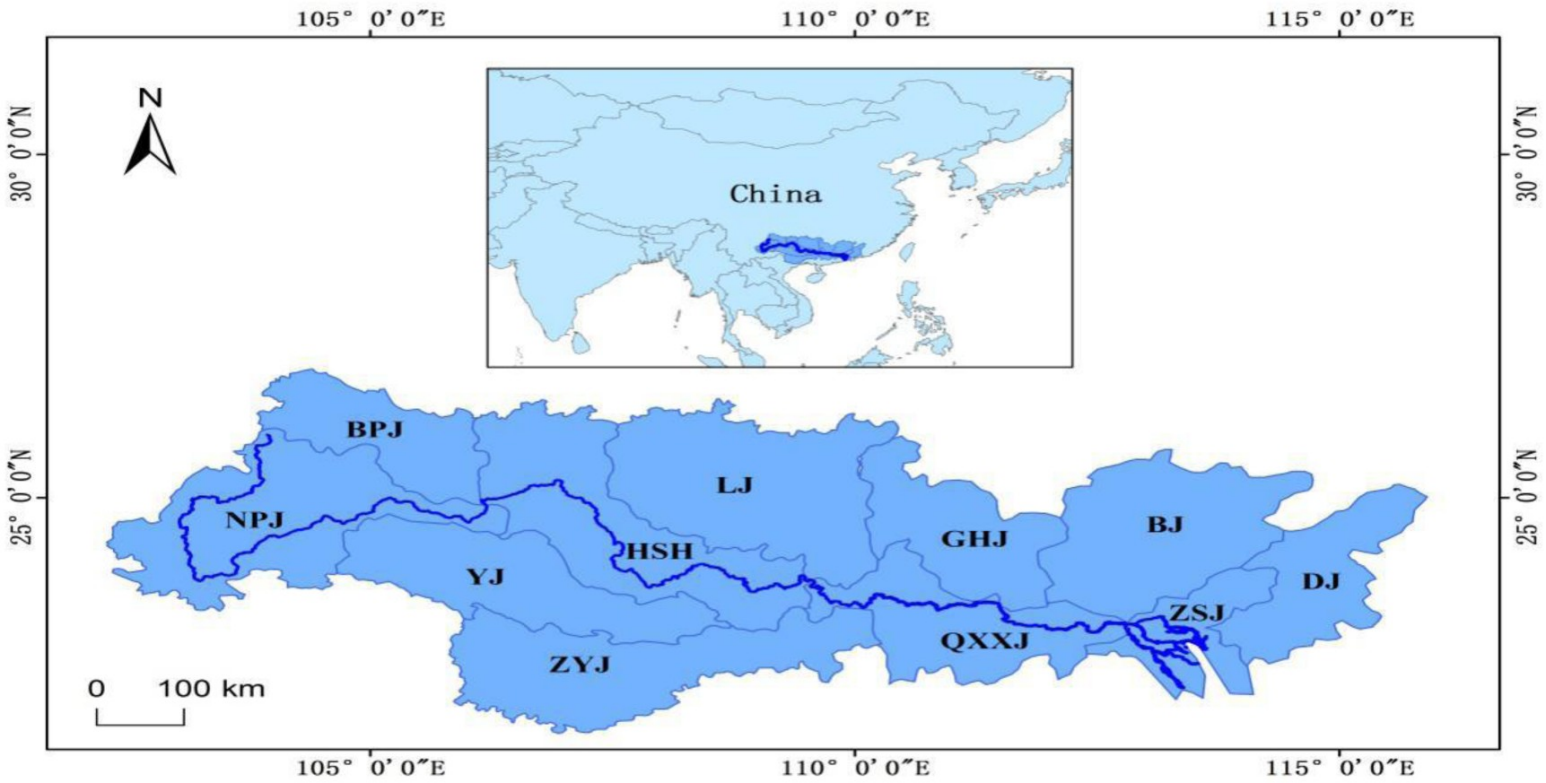
Geographical map of the Pearl River Basin and its sub-basins. NPJ -Nanpanjiang, BPJ-Beipanjiang, YJ -Youjiang, ZYJ-Zuoyujiang, HSH-Hongshuihe, LJ-Liujiang, GHJ-Guihe River, QXXJ-Qianxunxi River, BJ-Beijiang River, DJ -Dongjiang River, ZSJ-Pearl River Delta Basin. The map was created with ArcGIS 10.2, URL: http://www.esri.com/software/arcgis/arcgis-for-desktop. The Pear River Basin and its sub-basins were displayed according to previous study [37]. Other data of the map were obtained at the following web site: http://www.diva-gis.org/Data.

The Pearl River, located in the south China between 21°31′~26°49′*N* and 102°14′~115°53′*E*, is the second largest river and the third longest river in China. It originates from Wumen Mountain in the Yunnan-Guizhou Plateau and flows across seven provinces in central and western China, flowing into the South China Sea from eight downstream inlets (Fig 1). The total area of the Pearl River Basin is about 443,518 square kilometers (km^2^). According to the watershed characteristics, the whole watershed can be divided into 11 sub-basins through the ArcGIS platform [37], namely Nanpanjiang (NPJ), Beipanjiang (BPJ), Youjiang (YJ), Zuoyujiang (ZYJ), Hongshuihe (HSH), Liujiang (LJ), Guihe River (GHJ), Qianxunxi River (QXXJ), Beijiang River (BJ), Dongjiang River (DJ), and Pearl River Delta Basin (ZSJ) (Fig 1). The areas of these sub-basins range from about 17,335.20 km^2^ (ZSJ) to 58,776.42 km^2^ (NPJ). The map was created with ArcGIS 10.2, URL: http://www.esri.com/software/arcgis/arcgis-for-desktop. The Pear River Basin and its sub-basins were displayed according to Yan et al. 2014[37]. Other data of the map were obtained at the following web site: http://www.diva-gis.org/Data.

### 2.2 Data source

The Pearl River flows across seven provinces: Yunnan Province, Guizhou Province, Jiangxi Province, Guangxi Province, Hunan Province, Fujian Province, and Guangdong Province. In this study, NANI was estimated on 48 prefecture-level administrative units which covers 11 sub-basins[38, 39]. Data used to calculate NANI included four categories: social data (urban population, rural population), economic data (Gross Domestic Product-GDP, Gross Output Value of Agriculture -GOVA, and Gross Output Value of Industrial-GOVI. et al.), agricultural data (N fertilizer application, compound fertilizer application, agricultural product planting area and yield, and poultry stock) and energy consumption data (coal, crude oil, gasoline, diesel, fuel oil, and natural gas), a total of 36 index parameters. The above data was collected from Provincial Statistical Yearbooks, Provincial Statistical Yearbooks on Agriculture, Regional Statistical Yearbooks, and the China Statistics Yearbook for Regional Economy from 1986 to 2015 [40]. The socioeconomic data associated with these four components of the NANI calculation model in each sub-basin were obtained by combining that of the intersection of sub-basins and the related country-level units in terms of the area-weighted method on the ArcGIS platform.

Because of the changes of statistical indexes, some socioeconomic data (including energy consumption, fertilizer application, agricultural product planting area and yield, and poultry stock) at regional level in the early years were missing. We restored some missing data with statistical models and algorithms such as time series, regression models, and Expectation Maximization.

### 2.3 Estimation of four components of NANI

In general, NANI is composed mainly of four components: N fertilizer application, food/feed net N input, atmospheric N deposition, and agricultural N fixation [23, 41]. The estimating model of NANI is as follows,

$$NANI=N_{fert}+N_{food\&feed}+N_{atmos}+N_{crop}$$ (2.1)

Where NANI represents the Net Anthropogenic N Input; N_*fert*_ presents the N fertilizer application; N_*food&feed*_ represents the net food/feed N import; N_*atmos*_ represents the atmospheric N deposition; and N_*crop*_ represents the N fixation of crops. The unit for these four components is expressed in kilograms of N per square kilometer per year (kg N km^-2^ yr^-1^).

In order to calculate NANI, four N components were analyzed separately. However, they were different kinds of the statistical data adaptive to different sun-basin. In particular, the components of *N*_*crop*_ will depend on the kinds of planting crop in the region. The N input from fertilizers (N_*fert*_) was estimated based on the application amounts of N fertilizers and the N content in the compound fertilizers. The data of fertilizer N can be obtained directly from Regional Statistical Yearbooks, and the amount of compound fertilizer can be calculated with N fertilizers by multiplying a factor *r*_*n*_ (*r*_*n*_ = 35.71%) [42].

The N_*food&feed*_ was defined as the sum of human food and livestock feed N consumption [43]. Atmospheric N deposition is mainly composed of wet deposition and dry deposition; the ratio of dry to wet deposition of atmospheric N is about 3:7, therefore dry deposition can be obtained by calculating the wet deposition of atmospheric N [28, 44–46]. Crop N fixation (N_*crop*_) was obtained by multiplying the crop planting area and the N fixation rate per unit of crop planting area. According to previous literature, the N fixation rate of soybean is 9600 kg N km^-2^ yr^-1^, and peanuts is 8000 kg N km^-2^ yr^-1^, and rice is 3000 kg N km^-2^ yr^-1^, while other N fixation rates of dry land and gardens were regard as 1500 kg N km^-2^ yr^-1^ [14, 24].

The NANI in the entire Pearl River Basin was obtained by the area-weighted average of the NANI of each sub-basin, as follows:

$$NANI_{t,entire}=\sum_{i=1}^{11}\left({area}_{i}\cdot NANI_{t,i}\right)/{area}_{entire};t=1,2,\cdots ,30.$$ (2.2)

$${NANI}_{average.i}=\frac{1}{30}\sum_{t=1}^{30}{NANI}_{t,i}.$$ (2.3)

Here, *NANI*_*t*,*enti*re_ represents the NANI of the entire Pearl River Basin in the year *t*, *area*_*i*_ denotes the area of the sub-basin *i* for *i* = 1, …, 11; *NANI*_*t*,*i*_ denotes the NANI of sub-basin *i* in year *t*; *area*_*entire*_ denotes the total area of the Pearl River Basin, and *NANI*_*average*.*i*_ denotes the mean value of the NANI of sub-basin *i* across the thirty years examined in this study.

### 2.4 Pearson correlation test for the linear trend

We use Pearson correlation coefficient to measure the linear correlation between series *NANI*_*t*,*entire*_ and time series *t*, which is defined by

$$r=\frac{\sum_{t=1}^{30}\left(NANI_{t,entire}-{\overset{-}{NANI}}_{entire})(t-\overset{-}{t}\right)}{\sqrt{\sum_{t=1}^{30}{\left(NANI_{t,entire}-{\overset{-}{NANI}}_{entire}\right)}^{2}}\sqrt{\sum_{t=1}^{30}{\left(t-\overset{-}{t}\right)}^{2}}}$$

Where ${\overset{-}{NANI}}_{entire}=\frac{1}{30}\sum_{t=1}^{30}{NANI}_{t,entire}$ and $\overset{-}{t}=\frac{1}{30}\sum_{t=1}^{30}t$.

When the correlation coefficient *r* is positive, it shows that *NANI*_*t*,*entire*_ has a linear increase trend; in contrast, when *r* is negative, it shows that *NANI*_*t*,*entire*_ has a linear decrease trend. When using Pearson correlation test, if *r* value is larger than some critical value, the hypothesis *r* = 0 will be rejected, that is, it asserts that there exists linear trend; otherwise, it asserts that there does not exist linear trend.

## 3 Results and discussion

### 3.1 Spatial and temporal variations of NANI in Pearl River Basin

**Fig 2:**
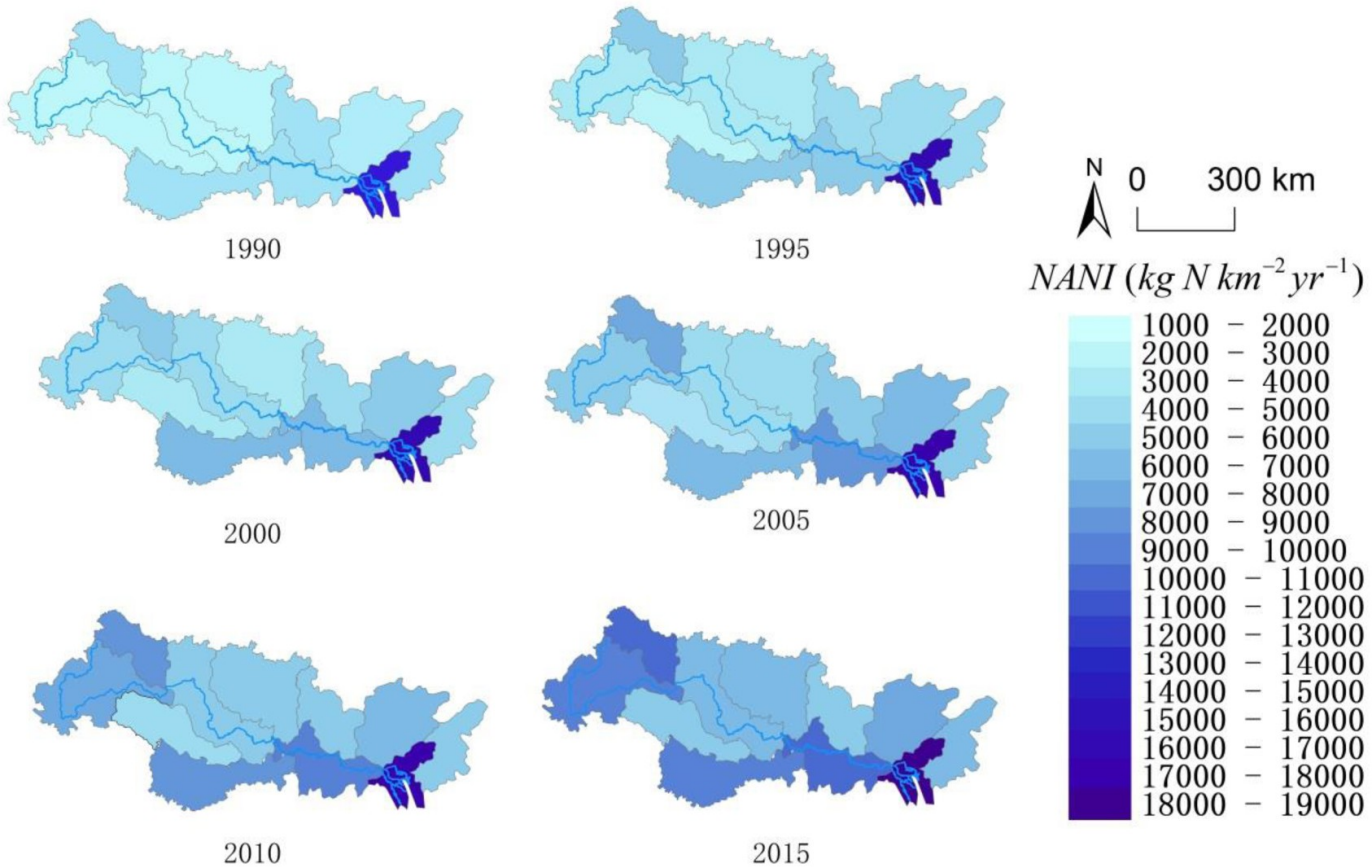
The temporal and spatial variations of NANI (kg N km^-2^ yr^-1^) in 11 sub-basins of the Pearl River Basin. These maps were created with ArcGIS 10.2, URL: http://www.esri.com/software/arcgis/arcgis-for-desktop. The Pear River Basin and its sub-basins were displayed according to previous study [37]. Other data of the map were obtained at the following web site: http://www.diva-gis.org/Data.

**Fig 3:**
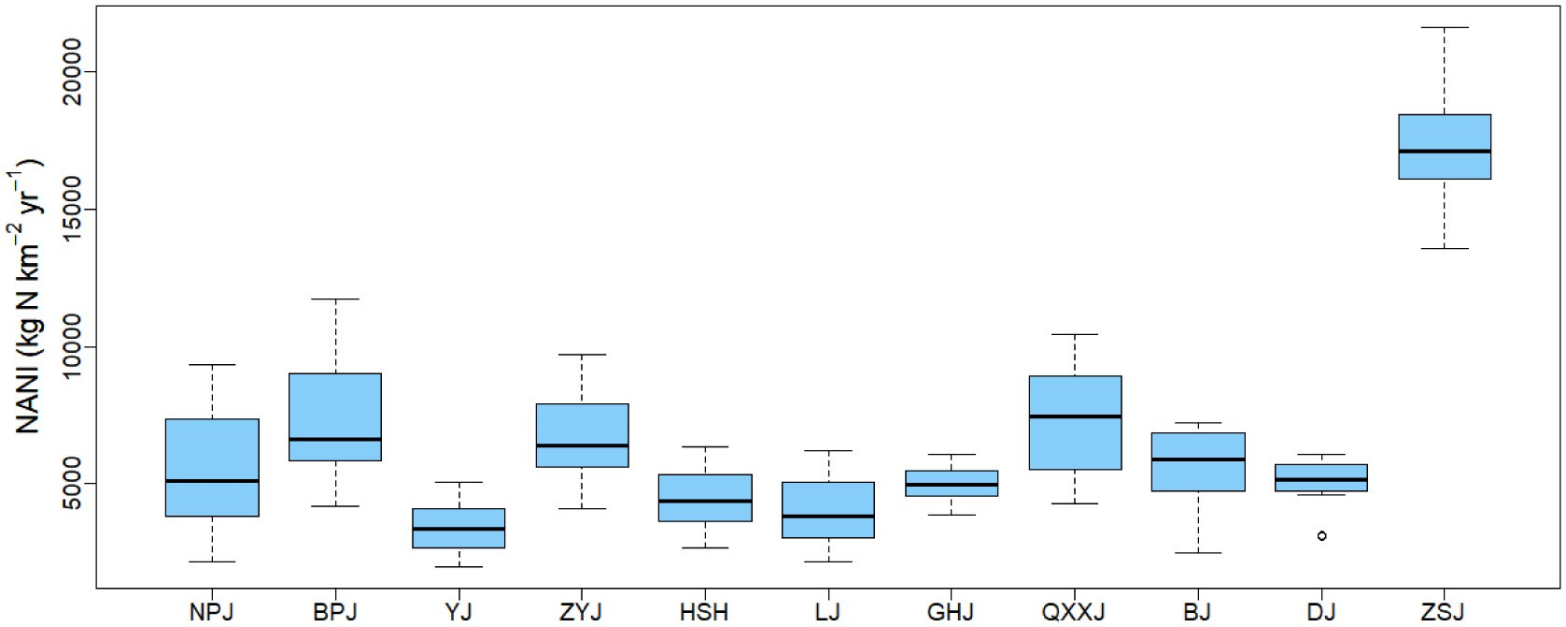
Box plot of multiannual average NANI in 11 sub-basins in the Pearl River Basin (1986–2015).

To examine the spatial differences in NANI in the Pearl River Basin, the multiannual (1986–2015) average NANI in each sub-basin was qualified. Figs 2 and 3 show the significant spatial distribution of NANI in 11 sub-basins. The greatest NANI was observed in the ZSJ sub-basin (16998.15 kg N km^-2^ yr^-1^), followed by the QXXJ (7416.16 kg km^-2^ yr^-1^) and BPJ (7410.04 kg km^-2^ yr^-1^) sub-basins, while the lowest NANI was recorded in the YJ sub-basin (3477.28 kg N km^-2^ yr^-1^). The spatial differences in the NANI distribution across the Pearl River Basin were related to the imbalance in the population distribution and economic development among the sub-basins (S1 and S2 Tables). In special cases, a significantly high NANI value in ZSJ was due to the dense population and industrial development in the joint region of the Xi, Dong and Bei rivers. NANI in Pearl River Basin has similar spatial pattern to that seen in the Yangtze River Basin [14], but it is relatively higher compared to other Basins in the world (western U.S. watersheds [26], northeastern U.S. watersheds [24, 25], Baltic Sea watersheds [27] and Indian watersheds [30]).

**Fig 4:**
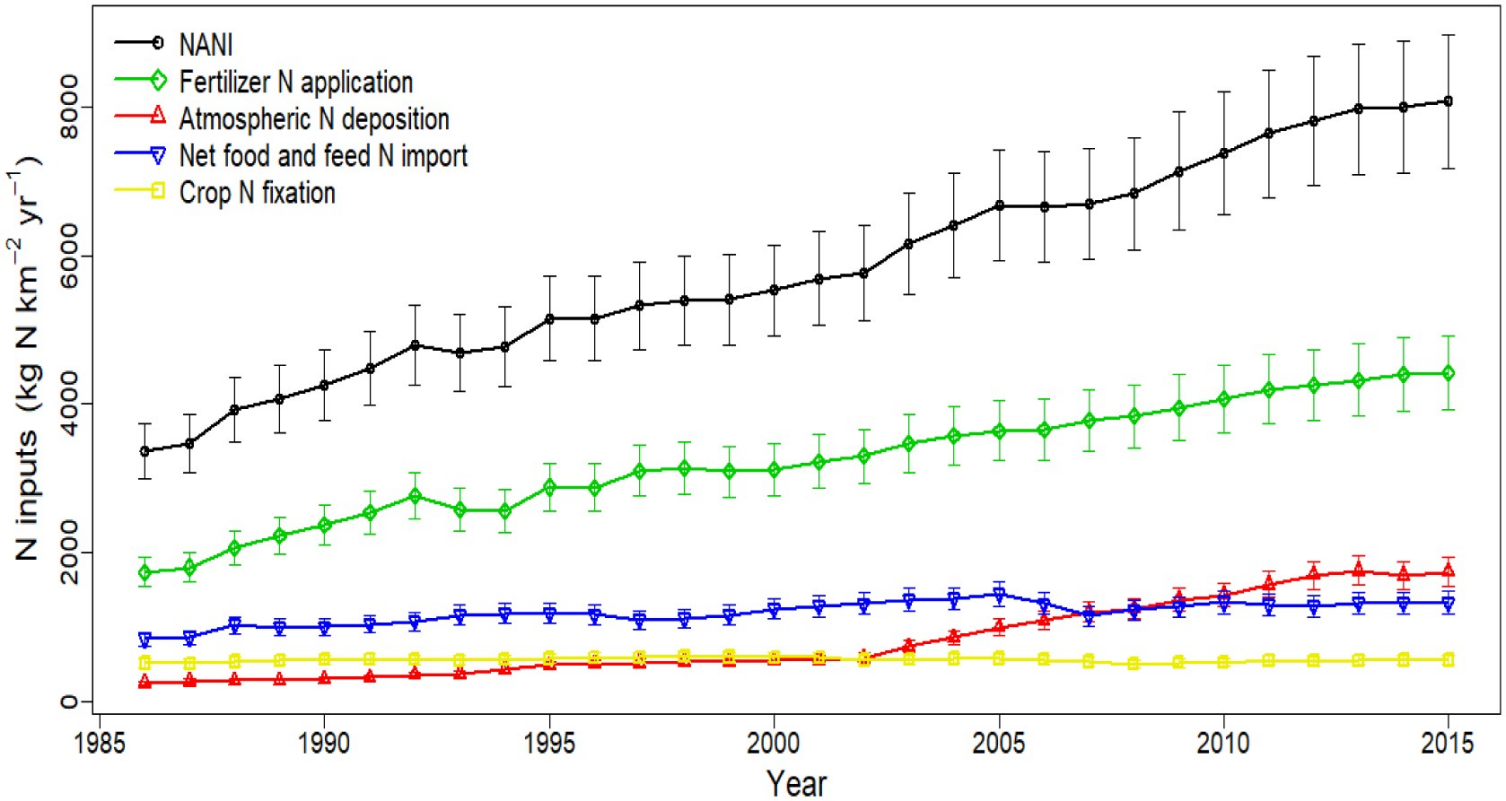
Total NANI and its four components in the Pearl River Basin from 1986 to 2015. The error bar denotes the 95% confidence interval of N inputs.

At same time, remarkable temporal shifts of NANI were observed in the Pearl River Basin (Fig 4 and S3 Fig), similar to the shift found in the Yangtze River basin[14]. The total NANI in the Basin gradually increased from 3,362.25 kg N km^-2^ in 1986 to 8,071.15 kg N km^-2^ in 2015, lower than the average of NANI (3388 kg N km^-2^ in 1990, 5013 kg N km^-2^ in 2009) in the same period in mainland China [29]. From 1986 to 2013, the total NANI increased at basically same rate of approximate 5% each year; after that, it showed a relatively slow increase during 2013–2015 (~1%). The increase of the total NANI in NPJ, BPJ, ZYJ and QXXJ was most significant among the sub-basins, which was mainly attributed to the rapid increase of fertilizer application in these regions. The N load in the Pearl River Basin has increased by about 2.5 times during the last three decades and the highest growth rate occurred in 1988 (13.1%) and the lowest growth rate occurred in 1993 (-2.1%). The temporal variation of each NANI component is shown in S1 Fig, indicating an increasing trend of fertilizer N application, atmospheric N deposition, and net food and feed N import during 1986 to 2015 (Pearson correlation test, *R*^*2*^ = 0.990, 0.953, 0.831, *p*<0.05), although there was a slight fluctuation in some years. On the contrary, Crop N fixation did not show a significant upward even downward trend from 1986 to 2015 (Pearson correlation test, *R*^*2*^ = -0.056, *p* = 0.778).

### 3.2 Contributions of different N sources to NANI in Pearl River Basin

The contributions of different sources to NANI in the Yangtze River Basin were quantified (Fig 4 and S2 Table). N fertilizer application was the main input source of NANI in the Pearl River Basin, increasing from 1,741.69 kg N km^-2^ in 1986 to 4,426.17 kg N km^-2^ in 2015, contributing 55.53% N to the total NANI. The largest input of fertilizer N was observed in the ZSJ sub-basin, though it showed a decreasing trend from 9750.85 kg N km^-2^ in 1986 to 6026.86 kg N km^-2^ in 2015, reflecting a decline of agricultural activities in the region over this period. Overall, N fertilizer use appeared to gradually increase over the period between 1986 and 2015, suggesting a continuous expansion of agricultural practices in most sub-basins. Food and feed net N import was the second largest source of NANI, and though its overall contribution dropped from 26.24% in 1988 to 16.55% in 2015 (mean = 21.26%), in the ZSJ, NPJ, and QXXJ sub-basins its contribution significantly increased during this period. Atmospheric N deposition contributed on average to 12.95% of the total NANI. Amongst the four components of NANI, atmospheric N deposition had the largest increase rate over time, from 252.68 kg N km^-2^ in 1986 to 1,760.54 kg N km^-2^ in 2015 with a growth rate of 596.75%. Its contribution to NANI grew significantly from 7.4% in 1986 to 21.51% in 2015, when it became the second largest source of N input into the Pearl River Basin. Crop N fixation had the lowest contribution to the total NANI, with a mean value of 10.26% and decreased over time from 14.23% in 1986 to 7.07% in 2015, especially in the ZSJ sub-basin; this suggests the total amount of crops decreased during this period.

Fertilizer N application accounted for more than 50% of the total amount of NANI, indicating that N fertilizer is a major contributing factor, while the similar results could be found in the previous studies [3, 14, 19, 30, 41, 47, 48]. The NANI from land sources into the Pearl River Basin during this period was 44.2 million (hm^2^), and the rice planting area in this region accounted for one fifth of the total planting area in China (China Statistical Yearbook, 2015) [40]. The input of fertilizer N in the rice planting system was relatively larger, and the loss was also relatively high [49, 50]. Agriculture also plays an important role in the Basin economy. The per capita cultivated area in the Pearl River Basin in 2015 was 0.062 (hm^2^), which was approximately 2/3 of the average in China (China Statistical Yearbook, 2015) [40]. Because of the high population (0.113 billion) in the Pearl River Basin, the area used for agriculture is relative large, and due to low efficiency fertilization practices, a large amount chemical fertilizer was lost from the farmland, then discharged into river water, and entered into estuarine and coastal waters at last. Over 1986 and 2015, the amount of fertilizer N was tripled from 1,741.69 kg N km^-2^ to 4,426.17 kg N km^-2^. The sub-basin with the highest average N fertilizer application was the Pearl River Delta, ranging from 5,137.97 kg N km^-2^ to 16,180.96 kg N km^-2^, while the sub-basin with the lowest average was the Youjiang River Basin, at 770.67 kg N km^-2^ to 3,104.04 kg N km^-2^. In the other Basins, previous studies have also shown that fertilizer N application was the most important source of N pollution in water bodies [41, 51–54].

### 3.3 The impact of GDP and population density on NANI

Several studies showed that human activities including industrial N fixation, food trade, energy consumption, and diet choice were significant drivers of N alteration in various scales [55–57]. The socioeconomic factors are often referred to as population density, gross domestic product and industrial structure and so on. According to the results of [13] and [58], population density plays a much more significant role for the changing of NANI compared to gross domestic product (GDP).

**Fig 5:**
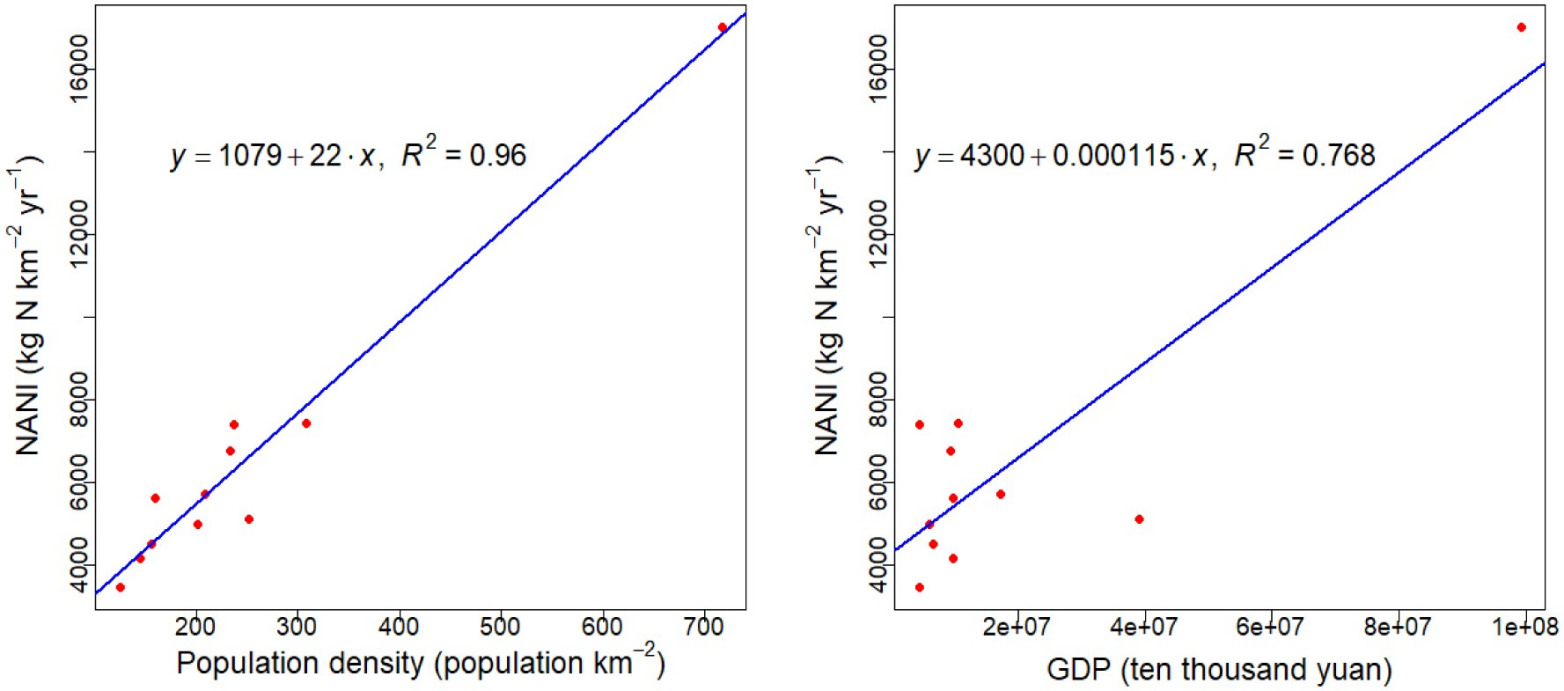
Correlations between NANI and regional GDP and population density over the 1990–2015 period.

S3 Table describes the main socio-economic features (average values across 1986–2015) in these 11 sub-basins, including area, population density, cultivated land area, gross domestic product, gross output of agriculture, and total grain output. Based on the main socio-economic data used in calculating NANI, the relationships between NANI and GDP, NANI and population density were analyzed. Fig 5 showed that GDP and population density were significantly linearly related to NANI. The slope of the linear regression between NANI and population density was 22, indicating that NANI will increase an average of 22 units with a one unit increase in population density. This result is similar to the result in previous studies in the Lake Taihu, Lake Dianchi and Lake Chaohu Basins [58]. The value of *R*^*2*^ of the linear regression between NANI and GDP was 0.768, indicating that approximately 76.8% of the total variation of NANI could be explained to the average. This is significantly higher than that reported in the previous study with an *R*^*2*^ of 0.31 in the Lake Taihu, Lake Dianchi and Lake Chaohu Basins[58], Therefore, population density and GDP should be important factors impacting the spatial variation of NANI in the area. Different distribution patterns of NANI across 11 sub-basins were resulted partly from the imbalanced population density and the development of industry and agriculture in these regions.

### 3.4 Impacts of NANI on red tide occurrence

**Fig 6:**
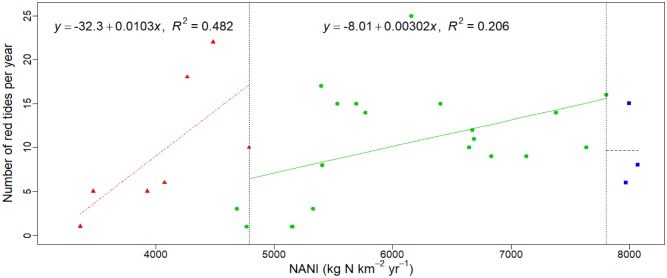
Correlation between NANI and the number of red tides occurring per year in Guangdong coasts between 1986–2015.

In recent decades, the occurrence of red tides has become increasingly frequent in the Pearl River Estuary and adjacent coastal areas [42, 59–61]. There were a total of 305 red tide events recorded in Guangdong coasts from 1986 to 2015. Among them, 29 times were in the western coast of Guangdong, 42 times were in the eastern coast, 53 times were in Daya Bay, 107 times were in Daopeng Bay, and 74 times were in Pearl River Estuary [61]. The annual frequency of red tides was generally less than 10 in the area before the 1980s, but there has been a significant increase of the frequency since the 1990s (18 times in 1990, 22 times in 1991, and 7 times in 1998). However, the annual frequency of red tides began to drop after the 2000s, remaining between 6 and 16 time per year except in 2003. The relationship between the NANI in the Pearl River Basin and the frequency of red tides in the Pearl River Estuary and the adjacent coast was shown in Fig 6. From 1986 to 1992, the frequency of red tides increased from 5 times per year to 15 times per year with the increase of NANI, with highest increase rate during the examined period. In the second period of 1993–2012, NANI had a poor linear correlation with the red tides (*R*^*2*^ = 0.206, *p* = 0.044), and the frequency of red tide had a significantly low increase rate. In the third period from 2013–2015, there was a slightly decrease in the frequency of red tide. In total, this analysis showed that NANI could promote the occurrences of red tide. When the NANI shifted from 1000 kg N km^-2^ yr^-1^ to 5000 kg N km^-2^ yr^-1^, the frequency of red tide increase most rapidly, suggesting N was relatively lack in the estuarine and coastal ecosystem. However, the N limit became weak with the increase of NANI over 5000 kg N km^-2^ yr^-1^, leading to the decrease of frequency of red tide in the second time period. When the NANI was up to 8000 kg N km^-2^ yr^-1^, N should be not a limiting factor for the occurrence of red tide. Together, this showed that it should be very important to control NANI in the Pearl River Basin to reduce the occurrence of red tide and protect the environmental health of the estuarine and coastal ecosystem. However, it will take a long time to reduce N loading substantially in China. For this purpose, some corresponding measures should be taken, for example strengthening the monitoring and forecasting of water quality in Pearl River Basin and the coastal, and adjusting the energy structure and so on.

## 4 Conclusion

The distribution of NANI in the Pearl River Basin has remarkable temporal and spatial variations. Over the past 30 years, NANI in the Pearl River Basin has increased with a relatively constant speed from the main four sources. N fertilizer application contributed the most to NANI input, followed by food/feed net N input, atmospheric N deposition, and crop N fixation. Over the last decades, atmospheric N deposition has significantly increased the second largest source of NANI, reflecting the rapid industrialization and urbanization in this region. Moreover, the increase of NANI in the Pearl River Basin was also strongly related to the increase in population and GDP. Statistical analysis showed the frequency of red tide in the Pearl River Estuary was promoted by NANI discharge (*R*^*2*^ = 0.90, *p* < 0.01). In total, this work provides a quantitative understanding of the temporal and spatial variations of NANI in the Pearl River Basin, which should be have significance for protecting the estuarine and coastal environment by reducing the input of NANI.

## Supporting information

S1 Table The proportion (%) of NANI input from different source in Pearl River Basin over 1986–2015 period. This map was created with ArcGIS 10.2, URL: http://www.esri.com/software/arcgis/arcgis-for-desktop. The Pear River Basin and its sub-basins were displayed according to previous study [37]. Other data of the map were obtained at the following web site: http://www.diva-gis.org/Data.
(DOCX)

S2 Table Analysis of variance (ANOVA) for the NANI components in the 11 sub-basins of the Pearl River Basin. Theses maps were created with ArcGIS 10.2, URL: http://www.esri.com/software/arcgis/arcgis-for-desktop. The Pear River Basin and its sub-basins were displayed according to previous study [37]. Other data of the map were obtained at the following web site: http://www.diva-gis.org/Data.
(DOCX)

S3 Table Areas and main socio-economic parameters (average values over 1986–2015) in 11 sub-basins of the Pearl River Basin. (DOCX)

S1 Fig Distribution of the four major net anthropogenic N inputs components between 1986 and 2015. (DOCX)

S2 Fig Distribution of the four major net anthropogenic N inputs components in 11 sub-basins in the Pearl River Basin during different period. 1990, 1995, 2000, 2005, 2010 and 2015 show the stage of 1986–1990, 1991–1995, 1996–2000, 2001–2005, 2006–2010 and 2011–2015 respectively.
(DOCX)

S3 Fig The temporal change of NANI in the Pearl River basin in three different sequential stages. (DOCX)

S1 Dataset The data relevant to the analysis for NANI over 1986–2015 period. (XLSX)

S2 Dataset The data relevant to the analysis for population density, GDP and red tide. (XLSX)
