# Supplementary material for: Temporal and spatial variations of net anthropogenic nitrogen inputs (NANI) in the Pearl River Basin of China from 1986 to 2015
Source: PLoS One. 2020 Feb 10;15(2):e0228683. doi: 10.1371/journal.pone.0228683 (PMC7010255; doi:10.1371/journal.pone.0228683)
Supplement: S1 Table — This map was created with ArcGIS 10.2, URL: http://www.esri.com/software/arcgis/arcgis-for-desktop. The Pear River Basin and its sub-basins were displayed according to previous study [37]. Other data of the map were obtained at the following web site: http://www.diva-gis.org/Data. (DOCX) [file pone.0228683.s001.docx]

**Supporting Information**

Table S1 The proportion (%) of NANI input from different source in Pearl River Basin over 1986-2015 period.

| Period | Fertilizer N application | Food and feed net N import | Atmospheric N deposition | Crop N fixation |
| --- | --- | --- | --- | --- |
| 1986-1990 | 53.46 | 24.91 | 7.40 | 14.23 |
| 1991-1995 | 55.94 | 23.75 | 8.37 | 11.94 |
| 1996-2000 | 57.22 | 21.70 | 9.93 | 11.16 |
| 2001-2005 | 56.14 | 22.25 | 12.16 | 9.45 |
| 2006-2010 | 55.69 | 18.24 | 18.33 | 7.74 |
| 2011-2015 | 54.72 | 16.70 | 21.51 | 7.07 |
| Annual average | 55.53 | 21.26 | 12.95 | 10.26 |
