# Supplementary material for: Temporal and spatial variations of net anthropogenic nitrogen inputs (NANI) in the Pearl River Basin of China from 1986 to 2015
Source: PLoS One. 2020 Feb 10;15(2):e0228683. doi: 10.1371/journal.pone.0228683 (PMC7010255; doi:10.1371/journal.pone.0228683)
Supplement: S2 Table — Theses maps were created with ArcGIS 10.2, URL: http://www.esri.com/software/arcgis/arcgis-for-desktop. The Pear River Basin and its sub-basins were displayed according to previous study [37]. Other data of the map were obtained at the following web site: http://www.diva-gis.org/Data. (DOCX) [file pone.0228683.s002.docx]

Table S2 Analysis of variance (ANOVA) for the NANI components in the 11 sub-basins of the Pearl River Basin.

| Variables | Sum of Squares | F-Statistics | *p* |
| --- | --- | --- | --- |
| N*_fert_* | 1028196418 | 71.257 | <2.2e-16*** |
| N*_atmos_* | 349519241 | 40.45 | <2.2e-16*** |
| N*_food&feed_* | 195254918 | 130.43 | <2.2e-16*** |
| N*_crop_* | 8630829 | 73.621 | <2.2e-16*** |

Note: ***, **, and* indicate the significance level of 0.1%s, 1%, 5%, 10% respectively.
