# Supplementary material for: Temporal and spatial variations of net anthropogenic nitrogen inputs (NANI) in the Pearl River Basin of China from 1986 to 2015
Source: PLoS One. 2020 Feb 10;15(2):e0228683. doi: 10.1371/journal.pone.0228683 (PMC7010255; doi:10.1371/journal.pone.0228683)
Supplement: S3 Table — (DOCX) [file pone.0228683.s003.docx]

Table S3 Areas and main socio-economic parameters (average values over 1986-2015) in 11 sub-basins of the Pearl River Basin.

| Sub-basins | Area (km^-2^) | Population Density (individual km^-2^) | Cultivated land area (km^-2^) | Gross Domestic Product (10^4^ RMB) | Gross Output of Agriculture (10^4^ RMB) | Total Grain Output (ton) |
| --- | --- | --- | --- | --- | --- | --- |
| NPJ | 58776.42 | 159.13 | 5163.07 | 9713283 | 2340845 | 2879306 |
| BPJ | 27542.37 | 236.79 | 3118.76 | 4440969 | 1068411 | 1771187 |
| YJ | 41843.02 | 124.57 | 4287.08 | 4361215 | 1492926 | 1527283 |
| ZYJ | 39714.29 | 233.78 | 6886.79 | 9301936 | 3125130 | 2776998 |
| HSH | 55514.73 | 155.35 | 5967.72 | 6590145 | 2225427 | 2498995 |
| LJ | 58369.25 | 144.21 | 5662.79 | 9822234 | 2380684 | 2646282 |
| GHJ | 32055.08 | 201.29 | 3358.04 | 6033929 | 2263787 | 2342631 |
| QXXJ | 36030.04 | 308.08 | 5009.02 | 10542327 | 3660200 | 3540768 |
| BJ | 47765.86 | 208.87 | 4754.65 | 17314824 | 3196228 | 3112127 |
| DJ | 28572.62 | 251.93 | 2272.60 | 39026858 | 1743975 | 1750854 |
| ZSJ | 17335.20 | 716.71 | 3028.40 | 99207878 | 3329882 | 1644773 |
