# Supplementary material for: Temporal and spatial variations of net anthropogenic nitrogen inputs (NANI) in the Pearl River Basin of China from 1986 to 2015
Source: PLoS One. 2020 Feb 10;15(2):e0228683. doi: 10.1371/journal.pone.0228683 (PMC7010255; doi:10.1371/journal.pone.0228683)
Supplement: S1 Fig — (DOCX) [file pone.0228683.s004.docx]

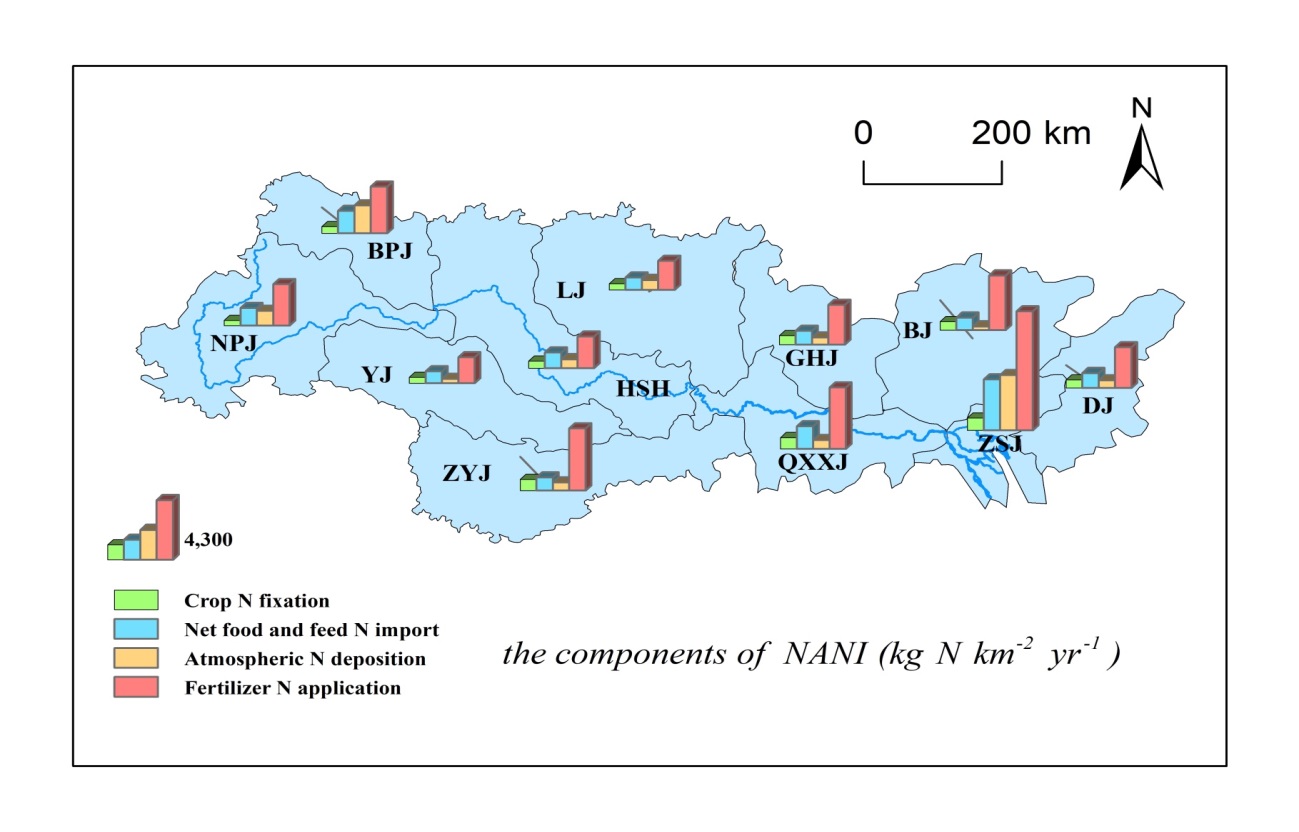


Figure S1 The proportion (%) of NANI input from different source in Pearl River Basin over 1986-2015 period. This map was created with ArcGIS 10.2, URL: http://www.esri.com/software/arcgis/arcgis-for-desktop. The Pear River Basin and its sub-basins were displayed according to previous study [37]. Other data of the map were obtained at the following web site: <http://www.diva-gis.org/Data>.
