# Supplementary material for: Temporal and spatial variations of net anthropogenic nitrogen inputs (NANI) in the Pearl River Basin of China from 1986 to 2015
Source: PLoS One. 2020 Feb 10;15(2):e0228683. doi: 10.1371/journal.pone.0228683 (PMC7010255; doi:10.1371/journal.pone.0228683)
Supplement: S2 Fig — 1990, 1995, 2000, 2005, 2010 and 2015 show the stage of 1986–1990, 1991–1995, 1996–2000, 2001–2005, 2006–2010 and 2011–2015 respectively. (DOCX) [file pone.0228683.s005.docx]

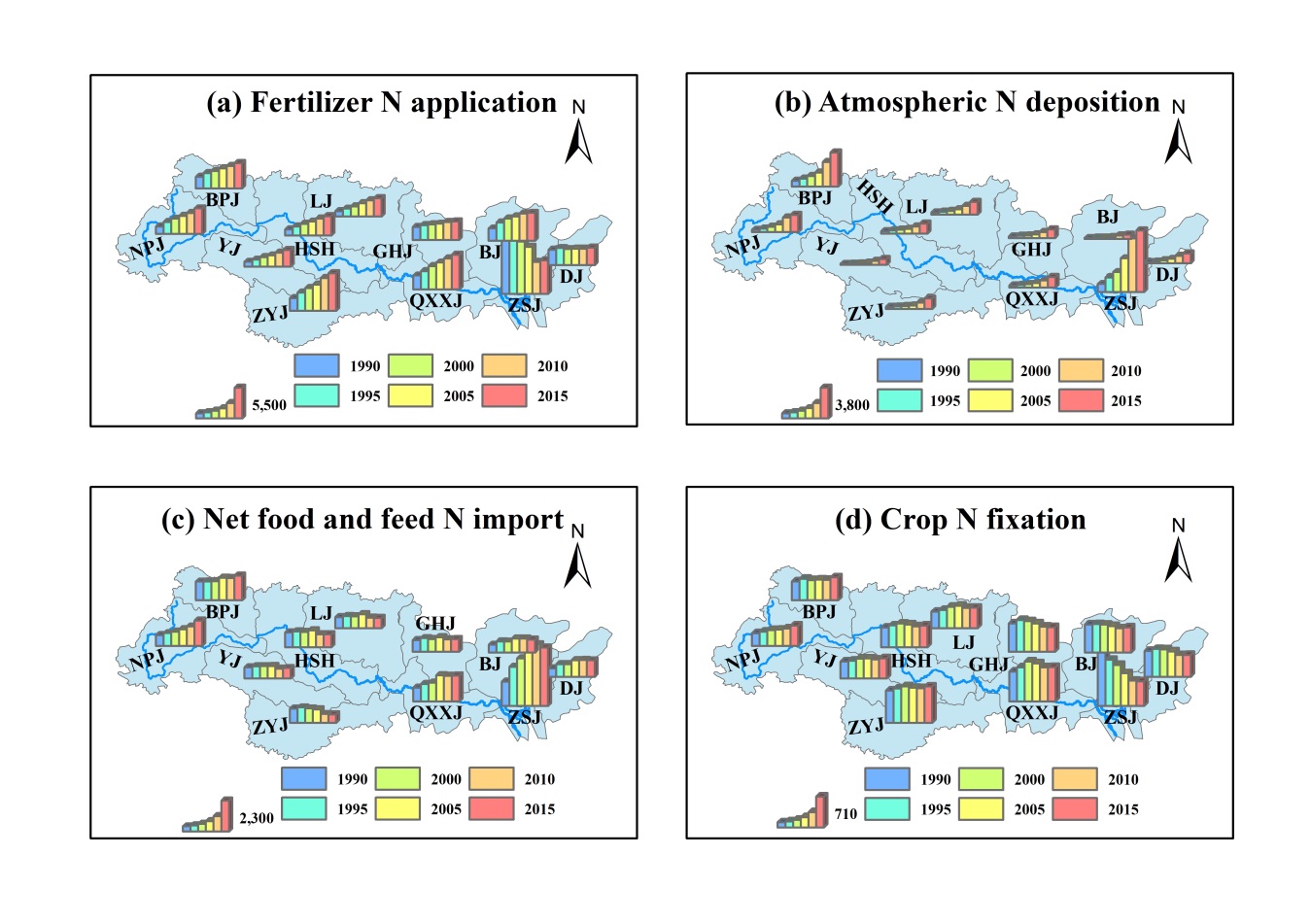


Figure S2 Analysis of variance (ANOVA) for the NANI components in the 11 sub-basins of the Pearl River Basin. Theses maps were created with ArcGIS 10.2, URL: http://www.esri.com/software/arcgis/arcgis-for-desktop. The Pear River Basin and its sub-basins were displayed according to previous study [37]. Other data of the map were obtained at the following web site: http://www.diva-gis.org/Data.
