# Supplementary material for: Temporal and spatial variations of net anthropogenic nitrogen inputs (NANI) in the Pearl River Basin of China from 1986 to 2015
Source: PLoS One. 2020 Feb 10;15(2):e0228683. doi: 10.1371/journal.pone.0228683 (PMC7010255; doi:10.1371/journal.pone.0228683)
Supplement: S3 Fig — (DOCX) [file pone.0228683.s006.docx]

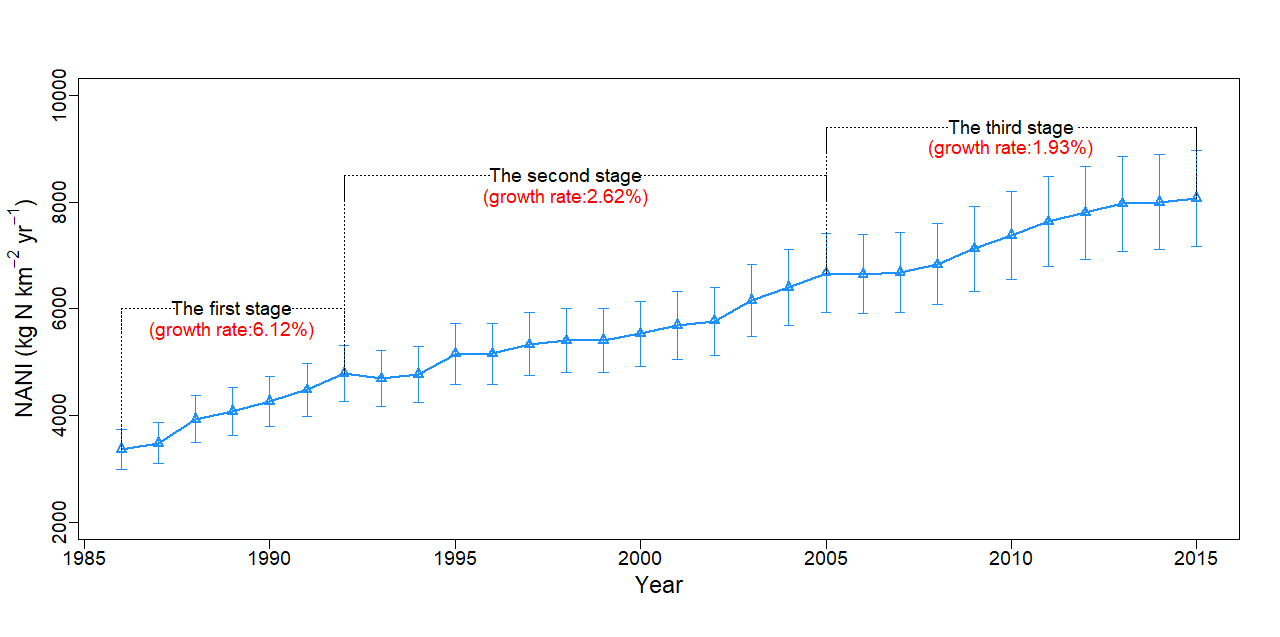


Figure S3 The temporal change of NANI in the Pearl River basin in three different sequential stages.
